# Metagenomic sequencing of bile from gallstone patients to identify different microbial community patterns and novel biliary bacteria

**DOI:** 10.1038/srep17450

**Published:** 2015-12-02

**Authors:** Hongzhang Shen, Fuqiang Ye, Lu Xie, Jianfeng Yang, Zhen Li, Peisong Xu, Fei Meng, Lei Li, Ying Chen, Xiaochen Bo, Ming Ni, Xiaofeng Zhang

**Affiliations:** 1 Department of Gastroenterology, Hangzhou First People’s Hospital, Hangzhou 310000, People’s Republic of China; 2 Department of Biotechnology, Beijing Institute of Radiation Medicine, Beijing 100850, People’s Republic of China; 3 Genomics Center of Academy of Military Medical Sciences, Beijing 100850, People’s Republic of China; 4 Department of Research Service, Zhiyuan Inspection Medical Institute, Hangzhou 310009, People’s Republic of China; 5 Department of Radiation Toxicology & Oncology, Beijing Institute of Radiation Medicine, Beijing 100850, People’s Republic of China

## Abstract

Despite the high worldwide prevalence of gallstone disease, the role of the biliary microbiota in gallstone pathogenesis remains obscure. Next-generation sequencing offers advantages for systematically understanding the human microbiota; however, there have been few such investigations of the biliary microbiome. Here, we performed whole-metagenome shotgun (WMS) sequencing and 16S rRNA sequencing on bile samples from 15 Chinese patients with gallstone disease. Microbial communities of most individuals were clustered into two types, according to the relative enrichment of different intestinal bacterial species. In the bile samples, oral cavity/respiratory tract inhabitants were more prevalent than intestinal inhabitants and existed in both community types. Unexpectedly, the two types were not associated with fever status or surgical history, and many bacteria were patient-specific. We identified 13 novel biliary bacteria based on WMS sequencing, as well as genes encoding putative proteins related to gallstone formation and bile resistance (e.g., β-glucuronidase and multidrug efflux pumps). Bile samples from gallstone patients had reduced microbial diversity compared to healthy faecal samples. Patient samples were enriched in pathways related to oxidative stress and flagellar assembly, whereas carbohydrate metabolic pathways showed varying behaviours. As the first biliary WMS survey, our study reveals the complexity and specificity of biliary microecology.

Cholelithiasis (gallstone disease) is prevalent in many regions of the world, and can occur at any place of the human biliary tract, including the gallbladder, extrahepatic duct or intrahepatic duct^1^. Ethnicity, gender, age, obesity, diet and lifestyle factors impact cholelithiasis morbidity, which varies between countries and regions^2^. In Western countries, cholesterol gallstones are highly prevalent (~75%), followed by black pigment stones (~20%)^3^. In Asia, the overwhelming majority of gallstones are brown pigment stones (BPSs), comprised of calcium bilirubinate, fatty acid soaps (e.g., calcium palmitate and calcium stearate) and mucin^3^.

Many studies have suggested a role of bacterial infection in gallstone pathogenesis^4^,^5^,^6^,^7^,^8^; however, a definitive causal relationship between biliary bacteria and gallstone formation has never been established. *De novo* formation of BPSs in bile ducts is generally thought to be related to biliary bacteria/parasite infection and partial biliary tract obstruction^2^,^9^. Many bacteria, such as *Escherichia coli*, *Klebsiella pneumoniae*, *Enterococcus faecium*, *Enterobacter cloacae* and *Pseudomonas aeruginosa*, have been identified in bile or gallstone samples through cultivation or polymerase chain reaction (PCR). Bacterially produced β-glucuronidase was suggested to be associated with the formation of pigment gallstones^4^,^10^. Other bacterial products, such as phospholipase and slime, might also participate in gallstone formation^6^.

Antimicrobial therapies are commonly used for biliary infections caused by gallstone disease, with bile cultures being used to select the appropriate antibiotic agent^11^,^12^. Furthermore, the Tokyo Guidelines for antimicrobial therapy of Acute Cholangitis, a common gallstone complication, proposed the use of bile cultures to aid in patient treatment^13^. However, as multiple bacteria coexist in the biliary tract, culture-dependent methods are somewhat insensitive and biased for bacterial identification and are inadequate to study the entire microbial community^9^,^14^.

Therefore, improved understanding of the biliary tract microbiota would be helpful for studies of bacteria-related gallstone pathogenesis and antibiotic therapies. Unlike conventional methods for identifying microorganisms, such as cultivation and bacteria-specific PCR, next-generation sequencing (NGS) provides a comprehensive picture of the microbial flora^15^,^16^. NGS has shown advantages for microbiome studies across multiple human body sites, including the gastrointestinal tract, oral cavity, nasal cavity, skin, vagina and placenta^17^,^18^,^19^. However, there have been few extensive NGS-based researches of the biliary tract. Wu *et al.* performed bacterial 16S rRNA amplicon sequencing (referred to as ‘16S sequencing’) on gallbladder bile, gallstones and faeces from cholesterol gallstone patients^16^. They detected many gut bacterial operational taxonomic units (OTUs) in the biliary tract and found that faecal samples from gallstone patients exhibited microbiota dysbiosis.

To the best of our knowledge, no study has used whole-metagenome shotgun (WMS) sequencing on biliary samples. Compared to 16S sequencing which employs bacterial universal primers, WMS sequencing has increased taxonomic resolution and provides insights into microbial functionality and biological processes^15^. Here, we used WMS and 16S sequencing to analyse bile samples of 15 Chinese patients with choledocholithiasis. Bile was collected from the common bile duct, where the gallstones *de novo* formed. To obtain an accurate and comprehensive picture of the biliary microbiota, we filtered host components within WMS data with stringent criteria, and constructed a large reference dataset including 9,671 bacterial genomes for bioinformatics analysis. We characterized the biliary microbial communities and compared them to those of gut flora. Thirteen novel biliary bacterial species with reliable WMS genome coverage were identified. Finally, we investigated genes possibly related to gallstone formation and bile resistance, and the metabolic network of the biliary microbiota was reconstructed.

## Results

### Microbial community heterogeneity among patients

**Figure 1:**
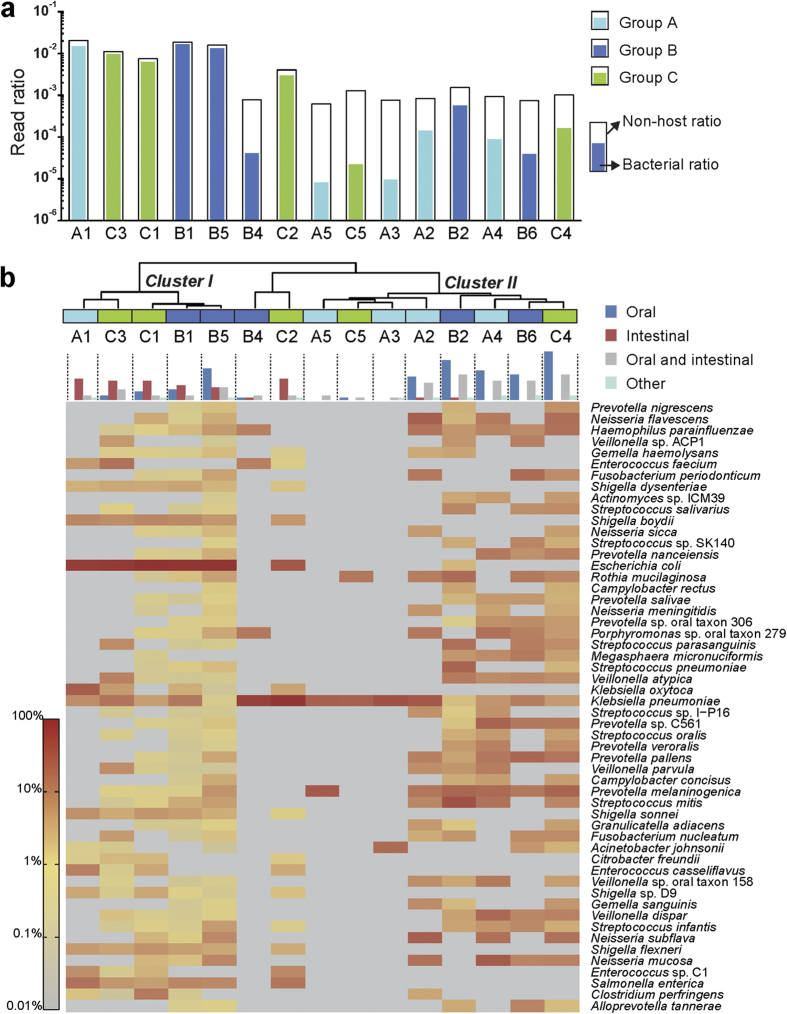
Distributions of WMS sequencing reads and microbial communities within bile samples. (**a**) Ratios of WMS sequencing reads after removing host background (hollow black-edged bars) and ratios of reads aligned to bacterial database (inner bars). All ratios were calculated by dividing by the total clean read number after quality control. Sample IDs are labelled on the x-axis, and ranked by their clustering relationships. Bacteria-aligned read ratios of samples from groups A, B and C are indicated by light blue, dark blue and green bars, respectively. (**b**) Hierarchical clustering of samples based on microbial community, distribution of bacterial origin and heatmap of species abundances. Individuals are denoted by coloured blocks, as in (**a**). Bacterial origins were classified as oral (referring to the oral cavity/respiratory tract), intestinal, both oral and intestinal, and other (environmental or unknown). For bacteria with ≥0.1% abundance in at least three individuals, the distribution of their origin in each individual is reflected by the histogram above the heatmap. The heatmap colour scale quantifies the log_10_ relative abundance of species, from grey (none or low abundance) to dark red (high abundance).

We performed unbiased WMS sequencing of total DNA extracted from 15 bile samples, obtaining an average of 7.29 Gbp per sample (range: 6.81–7.81 Gbp). WMS reads were filtered multiple times to remove host components, and then aligned to datasets containing 9,671 bacterial genomes from 3,608 species. Among these individuals, bacterial read percentages ranged from <0.001% to 1.66% (Fig. 1a). We did not find apparent between-group bias of bacterial ratios; that is, whether patients were febrile and/or had a history of endoscopic retrograde cholangiopancreatography (ERCP) was not correlated with the bacterial fraction in bile samples.

Bile microbial community analysis revealed heterogeneity among individuals. A total of 173 bacterial species were identified, and hierarchical clustering at species level divided these individuals into two major clusters (I and II) and one minor cluster (Fig. 1b; Supplementary Table S1). Clustering was not consistent with patient grouping. Interestingly, we found that oral cavity and respiratory tract inhabitants were more prevalent than intestinal inhabitants in the bile samples. Through literature mining, we defined 25 oral cavity/respiratory tract inhabitants, 11 intestinal inhabitants and 14 inhabitants of both sites among the 54 microbes observed in at least three patients with an abundance ≥0.1% (Supplementary Table S2). The 11 intestinal inhabitants were basically distributed in Cluster I (containing individuals A1, B1, B5, C1 and C3), which was highly enriched in *E. coli* (abundance range: 52.3–85.7%). Cluster I contained most of the intestinal microbes, whereas Cluster II (containing individuals A2–A5, B2, B6, C4 and C5) only contained two intestinal microbes. In particular, intestinal microbes such as *Shigella spp.* and *Salmonella enterica* were only detected in Cluster I and not found in Cluster II. Both major clusters contained 17 of the 25 oral cavity/respiratory tract inhabitants and 11 of the 14 microbes reported in both body sites. Samples in Cluster I consistently had higher bacterial read ratios (0.62–1.66%) than samples in Cluster II (0.0008–0.056%). Overall, the heterogeneity of the microbial communities can be attributed to the distribution of intestinal inhabitants, despite the presence of oral cavity/respiratory tract inhabitants.

Microbial species were frequently identified in only one (*n* = 77) or two individuals (*n* = 43) (Supplementary Table S1). High within-sample abundance was observed for some species, such as *Raoultella ornithinolytica* (15%) in A1, *Pyramidobacter piscolens* (3.39%) in C3 and *Proteus mirabilis* (0.6%) in C1. *K. pneumoniae* was the only species identified in almost all individuals (14/15).

### Bile microbial diversity revealed by WMS and 16S sequencing

**Figure 2:**
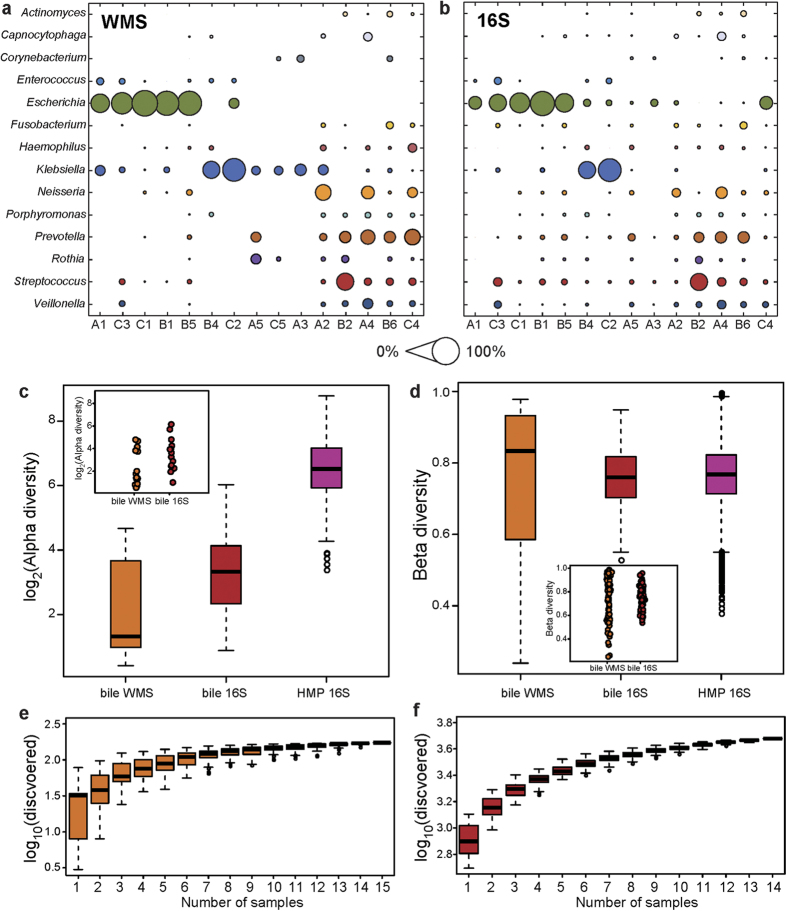
Microbial community characteristics generated by WMS and 16S sequencing. (**a–b**) Relative abundances at the genus level by WMS (**a**) and 16S sequencing (**b**). Circle sizes represent abundance, and circles are coloured by genus. Sample IDs are shown on the x-axis and are ranked as in Fig. 1. Common genera were those with a relative abundance ≥1% in at least three samples. The genera names for (**a**,**b**) are shown on the y-axis of (**a**). (**c–d**) Distributions of log_2_ alpha (**c**) and beta diversities (**d**). Orange and red boxplots denote distributions of WMS and 16S sequencing of bile samples respectively, accompanied with the distributions of faecal samples from HMP (purple boxplots). Log_2_ alpha and beta diversities are presented as scatter plots in insets (orange, bile WMS; red, bile 16S), with scales identical to the corresponding boxplot y-axis. (**e**–**f**) Species and OTU counts by WMS (**e**) and 16S sequencing (**f**) with increasing sample number. At a given sample number, a maximum 100-time random sampling of these bile samples was performed to calculate species or OTU counts. Boxes represent the interquartile range (IQR) between first and third quartiles (25th and 75th percentiles, respectively). Lines inside denote the median, and whiskers denote the most extreme values within 1.5 times IQR from the first and third quartiles, respectively. Outlier values are represented as circles.

Microbial communities determined by parallel 16S sequencing of 14 bile samples (Supplementary Table S3; DNA was unavailable for C5) generally agreed with communities determined by WMS sequencing, with 41 overlapping genera. Abundances of the genera exhibited a high correlation (Pearson correlation coefficient = 0.91) (Fig. 2a,b). For samples with insufficient bacterially aligned WMS reads (i.e., A5 and B4), 16S sequencing had greater sensitivity and could identify more genera. Although hierarchical clustering was not performed for 16S sequencing, the distributions of overlapping genera were somewhat similar (Fig. 2a,b), especially for highly abundant genera. For example, Cluster I was still enriched with intestinal genera (e.g., *Escherichia* and *Enterococcus*). However, 16S sequencing exhibited greater intra-sample differences in abundance (i.e., A5 and C4) than WMS sequencing, which could be attributed to the experimental biases of 16S sequencing.

Next, we compared the alpha and beta diversities of bile samples with those of healthy faecal samples (*n* = 319) from Human Microbiome Project (HMP)^17^,^18^ (Fig. 2c,d). As measured by inverse Simpson index, alpha diversities of bile samples based on WMS and 16S sequencing were significantly lower than those of faecal samples (Wilcoxon rank-sum test, *P* < 10^−8^). The difference in alpha diversity between bile WMS and 16S sequencing data (*P* = 0.029) might be due to differences in the sequencing strategy (Fig. 2c). On the contrary, there was no significant difference between the Bray–Curtis beta diversities of bile by 16S sequencing and faecal samples (*P* = 0.1753). Beta diversities of bile by WMS sequencing were borderline-significantly higher than those of bile by 16S sequencing (*P* = 0.044) and of faecal samples (*P* = 0.021). This result may be due, at least in part, to the varied sensitivity of WMS sequencing for bacterial identification. We also employed other alpha (Simpson index and Shannon index) and beta (Jaccard index) diversity indices to compare these samples, and obtained qualitatively consistent results (Supplementary Figs S1 and S2).

Accumulation curves of species and OTU counts are shown in Fig. 2e,f. Although bile samples exhibited less microbial diversity than faecal samples, the curves were not fully saturated. More samples and a more sensitive experimental design could be useful for enabling a more comprehensive analysis of the biliary microbiota.

### Newly identified microbes in bile

**Figure 3:**
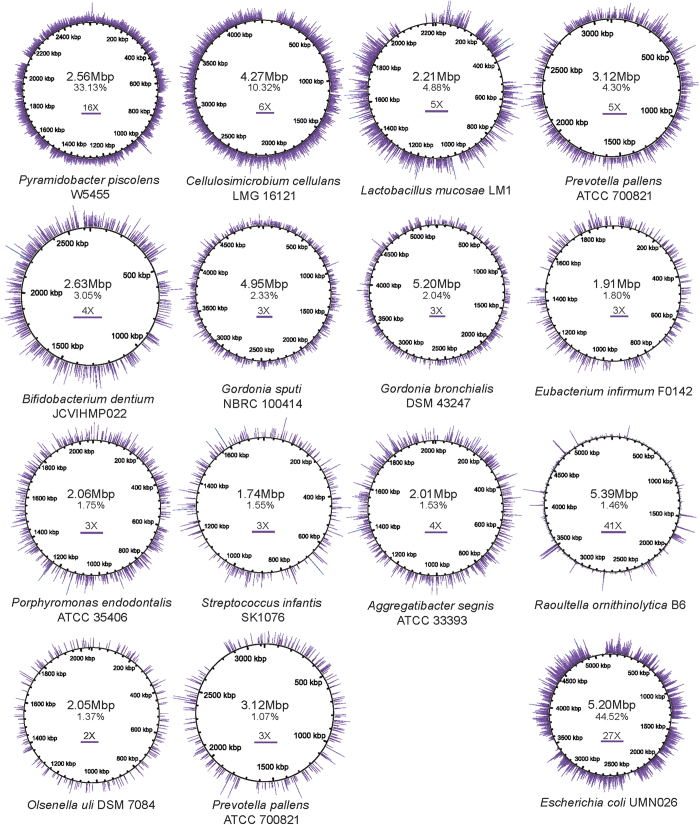
Genome coverage (≥1%) of bacteria newly identified in bile samples by WMS. Reference genomes of 13 newly identified bacteria are represented by circles generated by BLAST Ring Image Generator v0.95 (BRIG), with genome sizes labelled inside. Highly confident WMS read alignments (unique mapping and ≥99% identity) to these genome are illustrated by purple bars around circles. Bar height represents site coverage depth, with the corresponding scale indicated inside the circles. Overall coverage of the reference genome is labelled below the genome size. Newly identified bacteria are sorted by genome coverage. *P. pallens* ATCC 700821 appears twice because it was observed in two samples with ≥1% coverage (B5 and C4). *E. coli* UMN026, which has the highest WMS coverage (44%, in sample B5), is also shown at the end for reference.

Through WMS sequencing, we identified microbes that have not been reported previously in the human biliary tract. To ensure the reliability of taxon identification, we selected strain-specific WMS sequencing reads having ≥99% identity and full-length (100-bp) alignment with their reference genomes. We obtained 35 reference genomes with ≥1% coverage by these reads. Among these genomes were 13 microbe species that, to the best of our knowledge, have not previously been reported in bile samples (Table 1; Fig. 3). Nine of these species were individual-specific. In particular, *P. piscolens* and *Cellulosimicrobium cellulans*, identified in C3 and A1, respectively, exhibited ≥10% reference genome coverage. Five of the 13 novel species were also identified by 16S sequencing with ≥97% identity to species-level OTUs in Greengenes.

According to the Human Oral Microbiome Database^20^ and our literature investigation, eight of the 13 novel species were human oral microbial taxa; the rest were possible environmental taxa. We focused on the opportunistic pathogens. *P. piscolens* has been found in odontogenic abscesses, inflamed gingival crevices and infected canals^21^,^22^. *Olsenella uli* and *Porphyromonas endodontalis* are associated with human endodontic infections^23^,^24^. *C. cellulans* (strain LMG 16121), identified in A1 with a coverage of 10.32%, is an inhabitant of soil, water, decaying plant material and brewery sewage^25^. It rarely causes human infections, but may be involved in human septic arthritis^26^ and bacteraemia^27^. *R. ornithinolytica*, *Gordonia sputi* and *Gordonia bronchialis* are environmental bacteria that can cause bacteraemia^28^,^29^,^30^.

Other potentially novel microbe species in bile with < 1% reference genome coverage included opportunistic pathogens, such as *Streptococcus spp.*, *Prevotella spp.*, *Veillonella spp.*, *Neisseria spp.*, *Megasphaera micronuciformis* and *Gemella haemolysans*^31^,^32^,^33^,^34^,^35^, which often colonize the human oral cavity and upper respiratory tract. *Prevotella spp.* and *Veillonella spp.* are also inhabitants of the human gut. Many previously reported bacteria were also identified (Supplementary Table S4).

### Gallstone formation and bile resistance

We further investigated genes that might be related to gallstone formation or bile resistance. Previous studies have demonstrated potential associations of gallstone formation with bacterial products β-glucuronidase, phospholipase and urease^6^,^7^,^36^,^37^,^38^,^39^. Among the 173 bacterial species identified in the 15 bile samples, we extracted 34 species harbouring genes encoding these enzymes by searching the KEGG (Kyoto Encyclopedia of Genes and Genomes) GENOME Database. Using WMS sequencing, we also validated gene existence through metagenomic assemblies and metabolic reconstruction.

**Figure 4:**
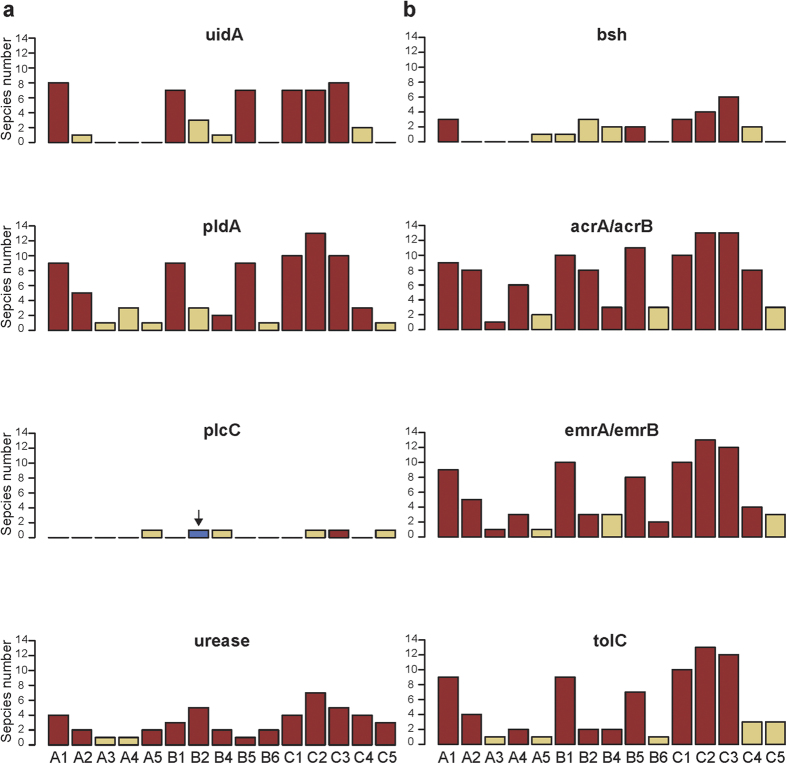
Distributions of bacterial species harbouring genes involved in gallstone formation (a) or bile resistance (b). Numbers of species within individuals are indicated by bars. Red bars denote genes that were also identified in metagenomic assemblies by gene prediction, or existed within metabolic reconstruction results. The gene encoding phospholipase C within individual B2 (blue bar with an arrow on top) was identified by metabolic reconstruction, but not by genome annotation. The term ‘urease’ on the left panel stands for genes encoding urease, which include ureA, ureB, ureC, ureD, ureE, ureF and ureG. The term ‘acrA/acrB’ stands for genes acrA and acrB, and ‘emrA/emrB’ stands for genes emrA and emrB.

β-glucuronidase and phospholipase hydrolyse biliary bilirubin and phosphatidylcholine, respectively, leading to precipitation of calcium bilirubinate and calcium palmitate and thereby facilitating pigment solid formation^6^. Seven and ten individuals had samples containing ≥3 species harbouring genes uidA (encoding β-glucuronidase) and pldA (encoding phospholipase A1), respectively (Fig. 4a; Supplementary Table S5). Samples from some individuals (i.e., A3, A5, B6 and C5) had one or no unvalidated related species. As the species numbers were correlated with bacterial WMS sequencing read ratios (Fig. 1a), failure to identify genes encoding β-glucuronidase or phospholipase might be due to issues related to DNA extraction or sequencing. For most individuals, the gene predictions did not identify the gene plcC encoding phospholipase C, which promotes cholesterol nucleation in human gallbladder bile^37^. *Helicobacter*-produced urease was reported to promote calcium precipitation, which might initiate gallstone formation^38^. Although the genes encoding urease were prevalent in the bile samples, *Helicobacter spp*. were not found among the urease-containing bacteria.

We also investigated genes involved in bile resistance (Fig. 4b). Bile salt deconjugation and multidrug efflux pump proteins are two mechanisms for bacterial bile resistance^8^,^40^. Within bile microbial communities in this study, we found 13 species with the gene encoding bile salt hydrolase (bsh) and 30 species with genes encoding efflux pump proteins (acrA, *n* = 29; acrB, *n* = 29; emrA, *n* = 23; emrB, *n* = 23; tolC, *n* = 22). Species harbouring bsh were less prevalent among individuals than species with efflux pump proteins, and the gene predictions were consistent with these observations.

Moreover, bacterial slime (i.e., glycocalyx) and biofilm play important roles in gallstone formation and bile resistance^6^,^41^,^42^,^43^. Strong slime producers, including *Prevotella intermedia*, *Clostridium perfringens*, *Enterococcus faecalis* and *E. faecium*, were identified in nine individuals at abundances of 0.1–7.99%^39^. High abundances (>0.5%) of biofilm producers, such as *E. coli*^44^, *P. aeruginosa*^45^, *P. intermedia*^39^ and *S. enterica*^43^, were also identified.

### Metabolic reconstruction of the biliary microbiota

**Figure 5:**
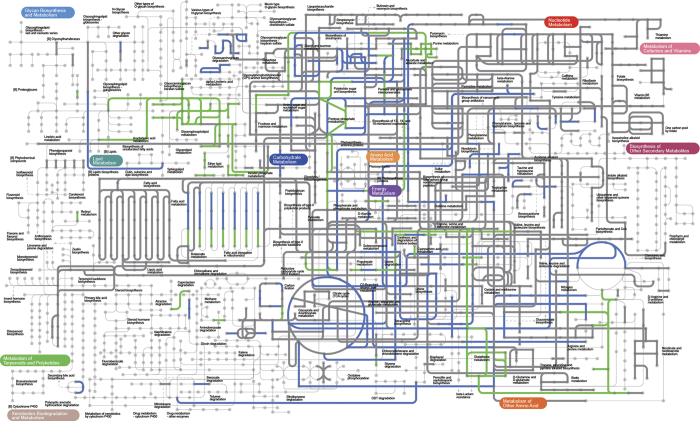
Metabolic reconstruction of the biliary microbiota. Bold grey lines represent abundant pathways that existed in at least five bile samples with ≥1% abundance. Pathways that are enriched or depleted in the biliary microbiota compared to healthy faecal microbiota from HMP are highlighted by green and blue lines, respectively.

Metabolic reconstruction was performed on the basis of the host-removed WMS reads (Fig. 5; Supplementary Table S6). Pathways involved in basic bacterial life dominated the bile microbiota metabolic network. Using predicted gene sets, we identified core eggNOG (evolutionary genealogy of genes: Non-supervised Orthologous Groups) functional categories, including carbohydrate transport and metabolism, amino acid transport and metabolism, transcription as well as energy production and conversion (Supplementary Table S7; Supplementary Fig. S3). When we projected eggNOG orthologous groups onto the KEGG pathways with iPath v2.0^46^ (Supplementary Fig. S4), we found that the overall distribution of pathways was similar to that of pathways identified by HUMAnN^47^ as shown in Fig. 5.

Next, we compared the metabolic networks of bile microbiota with those of faecal microbiota from HMP faecal samples (*n* = 136), focusing on differentially enriched pathways (Fig. 5; Supplementary Table S8). Bile samples were significantly enriched in pathways related to glycerophospholipid and glutathione metabolism (Wilcoxon rank-sum test, both adjusted *P* < 1.5 × 10^−4^), which are involved in inflammation and oxidative stress responses, respectively^48^,^49^. Upon investigating KEGG orthologues associated with resistance to oxidative stress (Supplementary Table S9), we observed an enrichment of glutathione reductase (NADPH, K00383, adjusted *P* < 2.1 × 10^−4^) and putative iron-dependent peroxidase (K07223, adjusted *P* < 0.042), consistent with the pathway analysis results. There was also an enrichment in the flagellar assembly pathway (adjusted *P* = 0.00022), which might be involved in biofilm development^45^ and bacterial sensory systems^50^. Glycolysis/gluconeogenesis, propanoate metabolism, ascorbate/aldarate metabolism, ABC transporter and phosphotransferase system pathways were also enriched (all adjusted *P* < 4 × 10^−5^).

There were many depleted pathways in bile samples compared to faecal samples (Fig. 5; Supplementary Table S8). For instance, the bile microbiota had decreased abundances of glycosaminoglycan and other glycan degradation pathways (both adjusted *P* < 1.3 × 10^−6^); this finding implies a decreased ability to employ glycan as a bacterial nutrition source^51^. Other depleted pathways included carbon fixation pathways in prokaryotes (adjusted *P* < 5.3 × 10^−7^) and amino acid metabolic pathways involving lysine, histidine, taurine and hypotaurine (all adjusted *P* < 2.9 × 10^−6^). Pentose phosphate pathway and starch/sucrose metabolism, which were related to carbohydrate metabolism, were also among the depleted pathways (both adjusted *P* < 2.4 × 10^−3^).

## Discussion

Asian countries, including China, have a high prevalence of BPS disease, which is the gallstone disease that is most closely associated with microbial infection. Thus, BPS disease could serve as a good model for gallstone-related bacterial studies^2^,^3^. Unexpectedly, we did not observe an apparent relationship between patient groups and microbial community features, as individuals from the same group had different patterns of biliary microbiota. Moreover, the bacterial read ratios of bile samples by WMS sequencing, which might reflect the bacterial load in the biliary environment, were not correlated with patient grouping. These results imply that the biliary microbiota might be shaped by more complicated factors, such as diet, lifestyle, host immune responses and/or specific anatomy structures, rather than by microbiota alone.

On the other hand, the lack of a correlation between patient grouping and microbial community may provide some insight into the clinical debate about ERCP procedures. Whether to perform endoscopic sphincterotomy (EST) or endoscopic papillary balloon dilation during ERCP remains controversial^52^,^53^, given concerns that EST could increase the risk of biliary tract infection^54^. We found that the microbial distribution of samples from choledocholithiasis patients who had no history of EST (groups A and B) did not differ greatly from the distribution of samples from afebrile patients who had a history of EST (group C). This observation implies that EST might not be a determining factor for the biliary microbial composition of BPS patients.

Our results were basically consistent between the WMS and 16S sequencing experiments, but had some discrepancies with the results of the investigation of Wu *et al.*^16^. Using 16S sequencing to study bile samples, these authors did not observe different biliary microbial community patterns among their subjects, and found that the bile samples had higher alpha diversities than faecal samples from healthy individuals and patients. The disagreement between their study and ours might be attributed to differences in gallstone type (cholesterol gallstones vs. BPSs), bile collection site (gallbladder vs. common bile duct), host and/or sequencing details (i.e., different 16S rRNA variable regions, V1–V2 vs. V3–V4) between the studies.

Compared to bacteria-specific 16S sequencing, biliary WMS reads were dominated by host components (>98%). Thus, the proportion of effective bacterial reads was relatively small, similar to those of the human vaginal introitus and mid-vagina^18^. Consequently, WMS sequencing exhibited less sensitivity to uncover less-abundant bacterial species, especially for samples with low bacterial ratios. Nonetheless, our recruited patients had distinct dominant species when either WMS or 16S sequencing was used. Moreover, even with limited data, WMS sequencing provided higher resolution for bacterial species identification and gene-level analysis. Finally, we reliably identified novel biliary bacteria by using a WMS sequencing analysis workflow with strict alignment thresholds.

WMS sequencing can be employed to obtain insights into microbial functionality. For example, information on gene abundance can be obtained by metagenomic assembly or direct alignment with protein databases. As this study is the first to apply WMS sequencing to bile samples, we paid special attention to genes encoding bacterial proteins involved in gallstone formation (e.g., β-glucuronidase, phospholipase and urease), which were identified by metagenomic assembly, metabolic reconstruction and/or KEGG GENOME annotation. The results agreed with previous reports and could be indicative of the roles of these genes in gallstone pathogenesis. Bile resistance-related genes, which could be crucial for bacterial survival, were also identified by the same methods. For example, although beneficial for microbial life in the biliary microenvironment, the role of bsh is not yet fully understood^8^. Genes encoding multidrug efflux pump proteins were more prevalent than bsh in our bile samples, suggesting that the former proteins might be more favourable for bacterial colonization and overgrowth in these patients.

To explore the biliary microbial functionality further, we studied the microbiota-host interplay at the pathway level. Bile acid and inflammation in the bile duct could both induce bacterial oxidative stress responses. Bacteria employ multiple pathways (e.g., cysteine/methionine, glutathione and riboflavin metabolism) to address oxidative stress^49^. Compared to HMP stool samples, patient bile samples were enriched in pathways related to glutathione metabolism and genes related to oxidative stress resistance (i.e., glutathione reductase). Another enriched gene, the putative iron-dependent peroxidase, is also involved in the oxidative stress response. These findings might reflect a mechanism by which the biliary microbiome maintains bacterial homeostasis and redox metabolism. Other oxidative stress-related pathways were not significantly enriched, implying that glutathione metabolism might be more crucial for microbial life in the biliary microenvironment. The observed increase in flagellar assembly suggests the possible enhanced motility of biliary inhabitants. Bile samples showed enrichment of pathways related to glycolysis/gluconeogenesis, propanoate metabolism and ascorbate/aldarate metabolism, but depletion of pentose phosphate and starch/sucrose metabolism pathways. These carbohydrate metabolic pathway results suggest a specific energy source and nutrition intake mode for the biliary microbiota.

Our study elucidates aspects of the biliary microbiota for patients with BPS disease. Nevertheless, the biliary microenvironment, microbial activity and their interplay with the host require further investigation. Specifically, to obtain a comprehensive picture of the biliary microbial composition and function, more ‘meta’omic’ (e.g., metagenomic, metatranscriptomic, metametabolomic and metaproteomic) datasets are required. Meta’omic studies could offer enormous potential for choledocholithiasis research and clinical practice.

## Conclusions

We applied WMS and parallel 16S sequencing methods to samples collected from the common bile ducts of Chinese choledocholithiasis patients. Microbial communities of these individuals were clustered into two categories: Cluster I, featuring species from the gut and oral cavity/respiratory tract, and Cluster II, featuring species from the oral cavity/respiratory tract. Given the direct connection between the bile duct and small intestine, biliary tract infections are generally believed to originate from retrograde infection of gut bacteria^55^,^56^. Surprisingly, we observed that oral cavity and respiratory tract inhabitants were more prevalent in bile samples than intestinal inhabitants. Thus, in addition to gut species, bacteria from the oral cavity/respiratory tract might be relevant to human biliary infection.

Using WMS sequencing, we identified 13 novel biliary bacterial species, which were of oral cavity (primarily) or environmental origin. Many patient-specific bacteria were found, implying the strong individuality of the biliary microbial community. We also identified putative genes related to gallstone formation and bile resistance. On the other hand, compared to HMP faecal samples from healthy individuals, the within-sample diversity of the bile microbial community was reduced, whereas the abundance levels of pathways involved in inflammation, oxidative stress response, flagellar assembly and membrane transport were enriched. Taken together, our results provide new insights into the biliary microbiota with respect to microbial composition and function, which could be valuable for clinical applications, such as the diagnosis and treatment of bile duct diseases.

## Materials and Methods

### Patients and sample collection

Bile samples were collected from the common bile ducts of 15 patients (mean age: 70.7 ± 16.8 years; 6 men, 9 women) who had been diagnosed with choledocholithiasis by computed tomography, magnetic resonance imaging, and B-mode ultrasonography at Hangzhou First People’s Hospital. All patients had BPSs in the common bile duct. There were no occurrences of gallbladder gallstones or hepatolithiasis. Patients were divided into three groups (*n* = 5 each), according to their fever status and/or history of ERCP: group A, febrile with no ERCP history (A1, A2, A3, A4 and A5); group B, afebrile with no ERCP history (B1, B2, B4, B5 and B6); and group C, afebrile with a history of ERCP with EST of Oddi’s sphincter (C1, C2, C3, C4 and C5).

A bile sample (2–5 mL) was collected from each patient during ERCP at Hangzhou First People’s Hospital, and the procedure of sample collection was sterilized (for details, see Supplementary Methods). Samples were immediately placed in germ-free sputum cups and stored at −80 °C until further processing. All patients provided written informed consent upon enrolment. The study conformed to the ethical guidelines of the 1975 Declaration of Helsinki and was approved by the Institutional Review Board of Hangzhou First People’s Hospital.

### DNA extraction from bile samples

For each patient, the total DNA was extracted from 400 μL of noncentrifuged bile sample by using the Invitrogen Purelink Genomic DNA Mini Kit (Life Technologies, Carlsbad, CA, USA), following manufacturer’s blood DNA extraction protocol. The DNA concentration was quantified with a Qubit 2.0 Fluorometer (Life Technologies), and quality was examined with the E-Gel electrophoresis system (Life Technologies).

### WMS sequencing

Total DNA from bile samples was sheared into ~350-bp fragments by the Covaris M220 Focused-ultrasonicator (Covaris, Woburn, MA, USA). Multiplexed libraries were prepared by using TruSeq Nano DNA Sample Preparation Kits (Illumina, San Diego, CA, USA) according to manufacturer’s instructions. Illumina HiSeq2500 platform was used to generate 2 × 100-bp pair-end sequencing reads.

### 16S sequencing

A two-step PCR protocol for 16S rRNA gene amplification and library preparation was used to perform 16S sequencing on Illumina MiSeq platform. The detailed protocol is listed in Supplementary Methods.

### Removing host reads from WMS sequencing data

Low-quality WMS reads (having >50% of bases with a quality <Q20 or having >10 N bases) were removed. Then, clean reads were aligned to the human genome (version 38) and to human sequences deposited in the National Center for Biotechnology Information (NCBI) Nucleotide Database (770,558 sequences) by BWA v0.7.5a^57^ and BMTagger (ftp://ftp.ncbi.nlm.nih.gov/pub/agarwala/bmtagger), both with default parameters. Unaligned reads were aligned again to human sequences in the NCBI Nucleotide Database with BLASTN v2.2.28+ (word size = 20, identity ≥80%, alignment length ≥80 bp, other parameters as default). Reads that failed these alignments were used for further analysis.

### Taxonomic analysis of host-removed WMS sequencing data

We downloaded 2,773 complete and 6,898 draft bacterial genomes from NCBI (Jul-Aug 2014). Host-removed WMS reads were aligned to these genomes by BLASTN (parameters: perc identity = 85, word size = 16, max target seqs = 30, outfmt = 6, evalue = 0.0000001, dust = no). Alignments were further filtered by using the following criteria: (1) sequence identity ≥90%, (2) alignment length ≥80 bp, (3) paired-end reads align to the same reference genomes, and (4) one reference genome should have reads that uniquely mapped to it. Abundances of reference genomes were calculated as described^58^ and normalized by genome size. For each individual, the abundances of the reference genomes were scaled to sum to one (i.e., to calculate the corresponding relative abundance). Relative abundances of species, genus and phylum were calculated by adding the relative abundances of all their taxonomic members deposited in the NCBI Taxonomy Database. Species with ≥0.1% relative abundance in at least one individual was considered for further analysis.

### Taxonomic analysis of 16S sequencing data

For details on quality control, chimera removal, OTU clustering and taxonomy assignment of 16S sequencing data, see Supplementary Methods.

### Hierarchical clustering of microbial communities

Microbial communities of samples were hierarchically clustered and visualized by the R language (version 3.1.2) gplots package (heatmap.2 function). Z-scores for microbes were calculated based on the microbial abundance distributions. Then, hierarchical clustering was performed with the complete linkage method based on the Euclidian distance between Z-scores for all possible sample pairs.

### HMP data retrieval

The OTU table generated by 16S sequencing and the corresponding metadata were downloaded from http://hmpdacc.org/HMQCP. Metabolic profiles by WMS sequencing, including metadata, were downloaded from http://hmpdacc.org/HMMRC. The procedure to analyse these 16S and WMS sequencing datasets was described previously^17^,^18^. Datasets of stool samples from healthy individuals were extracted for downstream analysis.

### Microbial diversity index

Inverse Simpson and Bray–Curtis dissimilarity indices were used to calculate the within-sample alpha diversity and the between-sample beta diversity, respectively. Both indices were measured by 16S OTU counts or species-aligned WMS reads. The R language vegan package was employed for these calculations.

### Metabolic reconstruction and functional annotation

For details on metabolic reconstruction, gene prediction and annotation, see Supplementary Methods.

### Statistical analysis

Correlations between the overlapping genera of the WMS and 16S sequencing were identified by using Pearson’s correlation in the R language. Wilcoxon rank-sum test (R v3.1.2) was employed to detect differences between the bile and HMP faecal samples with respect to microbial diversities and KEGG profiles. Under the condition of multiple comparisons, *P*-values were corrected to control the false-discovery rate by using the method described previously^59^. Differences with *P* < 0.05 or adjusted *P* < 0.05 were considered to be statistically significant.

## Additional Information

**Accession codes**: All WMS and 16S sequence data were deposited in the NCBI database under BioProject PRJNA278310. All bile samples were registered in the NCBI database under BioSample numbers SAMN03417491–SAMN03417505.

**How to cite this article**: Shen, H. *et al.* Metagenomic sequencing of bile from gallstone patients to identify different microbial community patterns and novel biliary bacteria. *Sci. Rep.* **5**, 17450; doi: 10.1038/srep17450 (2015).

## Supplementary Material

Supplementary Information

Supplementary_Table_S1

Supplementary_Table_S3

Supplementary_Table_S5

Supplementary_Table_S6

Supplementary_Table_S7

Supplementary_Table_S8

Supplementary_Table_S9

## Figures and Tables

**Table 1:** Bacteria newly identified within bile samples by WMS/16S sequencing, ranked by WMS genome coverage.

Species	Sample No.	Genus in 16S	Species in 16S^a^	WGS Genome Coverage^b^
*Pyramidobacter piscolens*	1	Yes	Yes	33.13%
*Cellulosimicrobium cellulans*	1	Yes	No	10.33%
*Lactobacillus mucosae*	1	Yes	Yes	4.88%
*Prevotella pallens*	6	Yes	Yes	4.30%
*Bifidobacterium dentium*	1	Yes	No	4.05%
*Gordonia sputi*	1	Yes	No	2.33%
*Gordonia bronchialis*	1	Yes	No	2.04%
*Eubacterium infirmum*	1	No	No	1.80%
*Porphyromonas endodontalis*	2	Yes	Yes	1.75%
*Streptococcus infantis*	5	Yes	No	1.55%
*Aggregatibacter segnis*	2	Yes	Yes	1.53%
*Raoultella ornithinolytica*	1	No	No	1.46%
*Olsenella uli*	1	No	No	1.37%

^a^Species-level OTUs required ≥97% sequence identity with Greengenes references.

^b^For species in multiple bile samples, the highest coverage value is shown.
