# Supplementary material for: Metagenomic sequencing of bile from gallstone patients to identify different microbial community patterns and novel biliary bacteria
Source: Sci Rep. 2015 Dec 2;5:17450. doi: 10.1038/srep17450 (PMC4667190; doi:10.1038/srep17450)
Supplement: Supplementary Information [file srep17450-s1.pdf]

Supplementary Information

Metagenomic sequencing of bile from gallstone patients to identify different microbial community patterns and novel biliary bacteria

Hongzhang Shen[1*], Fuqiang Ye[2,3*], Lu Xie[1], Jianfeng Yang[1], Zhen Li[2,3], Peisong Xu[4], Fei Meng[4], Lei Li[4], Ying Chen[5], Xiaochen Bo[2,3†], Ming Ni[2,3†] & Xiaofeng Zhang[1†]

[1]Department of Gastroenterology, Hangzhou First People's Hospital, Hangzhou 310000, People's Republic of China
[2]Department of Biotechnology, Beijing Institute of Radiation Medicine, Beijing 100850, People's Republic of China
[3]Genomics Center of Academy of Military Medical Sciences, Beijing 100850, People's Republic of China
[4]Department of Research Service, Zhiyuan Inspection Medical Institute, Hangzhou 310009, People's Republic of China
[5]Department of Radiation Toxicology & Oncology, Beijing Institute of Radiation Medicine, Beijing 100850, People's Republic of China

[†]Corresponding authors: Xiaochen Bo (email: boxc@bmi.ac.cn), Ming Ni (email: ni.ming@163.com), or Xiaofeng Zhang (email: zxf837@tom.com)

Supplementary information includes:
Supplementary methods, Supplementary Figures S1 to S4, Supplementary Tables S2 and S4.

Supplementary methods

ERCP Procedures. ERCP was performed by using side-viewing endoscopes (TJF240/JF-260V; Olympus Optical, Tokyo, Japan) which were strictly sterilized. In the process of putting endoscope from mouth into duodenum before suction of bile, the work channel of endoscope kept itself clean by avoiding pumping action. Sterile sphincterotome catheter (V-SYSTEM; KD-V411M-0725; Olympus Optical) which passed through the work channel was used to suck out bile sample (2–5 mL) from the common bile duct, before injection of contrast. Sphinceterotome catheter was also utilized to perform cannulation of Oddi's sphincter for patients who received ERCP for the first time. Bile samples were immediately placed in sterile sputum cups and stored at -80 ℃ until further processing.

All ERCPs were performed by the same endoscopist, who has considerable experience, based on the performance of more than 1,000 biliary interventions per year. Fellows or residents were involved only in the preparation of the patients before ERCP or helping the endoscopist during the procedure.

16S sequencing. Universal primer pairs 356F (5'CCTACGGGNGGCWGCAG3') and 803R (5'GACTACHVGGGTATCTAATCC3') targeting the V3-V4 region of the bacterial 16S rRNA gene were used for amplification. The two-step PCR protocol used by Ottesen *et al.*[1] was employed for 16S rRNA gene amplification and library preparation. In the first step, the target region of 16S rRNA was amplified by using 16S primers with attached overhang adapters (forward primer overhang adapter 5'TCGTCGGCAGCGTCAGATGTGTATAAGAGACAG3', reverse primer overhang adapter 5'GTCTCGTGGGCTCGGAGATGTGTATAAGAGACAG3') compatible with the Illumina Nextera DNA indices. Then, P5/P7 adapters and sample barcodes in the Nextera XT Index Kit (Illumina) were added to the cleaned-up PCR products in the second-round PCR. The final PCR products were cleaned up and sequenced

by the Illumina MiSeq platform to generate 2 × 250-bp paired-end reads covering the amplified 16S V3-V4 region.

Taxonomic analysis of 16S sequencing data. Pair-end reads generated by 16S sequencing were merged by using FLASH v1.2.11[2] to obtain ~460-bp V3-V4 16S sequences. Merged reads were analysed with QIIME v1.8.0[3]. First, reads with N bases were removed. Reads containing three or more consecutive low-quality bases ($Q < 20$) were truncated and the length of a trimmed read should be ≥75% of its original length. Reads passing the quality filter were aligned to the Greengenes Database (version Aug, 2013) for chimera check by USEARCH v6.1. Then, UCLUST[4] was applied for OTU clustering at the 97% similarity level. Ribosomal Database Project classifier v2.2[5] was retrained with the Greengenes Database and utilized to assign taxonomic rank to each representative OTU.

Function, pathway and metabolic annotations

Metabolic reconstruction. Host-removed WMS reads were aligned to the KEGG database (FTP release 13 Dec 2013) by MBLASTX (MulticoreWare, St. Louis, MO). Up to 20 hits (E-value $< 10^{-5}$) of each read were retained and analysed by HUMAnN v0.99[6]. Relative abundance and coverage values of the KEGG pathways and modules, and the KEGG orthology abundance values were calculated. KEGG pathways identified in at least five samples with >0.01 relative abundance were displayed by using iPath 2.0[7].

Gene prediction and annotation. Host-removed WMS reads were employed for *de novo* assembly with Velvet v1.2.0[8] and Metavelvet v1.2.02[9]. Reads were assembled into contigs with a minimum size of 300 bp and coverage cutoff of 2. The series of k-mer values were tested and the one maximizing the N50 value was

chosen. Genes were predicted from the contigs by using MetaGeneMark (prokaryoticGeneMark.hmm version 2.8)[10]. Redundant genes were removed if they shared ≥95% sequence identity over 90% of the shorter gene size in pairwise alignments by Usearch v7.0.1090. Relative abundances of genes were calculated as described[11].

Protein sequences of each non-redundant gene set were aligned to eggNOG v4 by using NCBI BLASTP (E-value $\leq 10^{-5}$). Genes having the highest scoring hit(s) with a bit score > 60 were assigned to the corresponding eggNOG orthologous group. The abundance of an eggNOG orthologous group was calculated by adding the abundances of genes assigned to the group.

References

1 Ottesen, A. R. *et al.* The impact of systemic and copper pesticide applications on the phyllosphere microflora of tomatoes. *J. Sci. Food Agric.* **95**, 1116-1125 (2015).

2 Magoč, T. & Salzberg, S. L. FLASH: fast length adjustment of short reads to improve genome assemblies. *Bioinformatics* **27**, 2957-2963 (2011).

3 Caporaso, J. G. *et al.* QIIME allows analysis of high-throughput community sequencing data. *Nat. Methods* **7**, 335-336 (2010).

4 Edgar, R. C. Search and clustering orders of magnitude faster than BLAST. *Bioinformatics* **26**, 2460-2461 (2010).

5 Cole, J. R. *et al.* The Ribosomal Database Project: improved alignments and new tools for rRNA analysis. *Nucleic Acids Res.* **37**, D141-D145 (2009).

6 Abubucker, S. *et al.* Metabolic reconstruction for metagenomic data and its application to the human microbiome. *PLoS Comput. Biol.* **8**, e1002358 (2012).

7 Yamada, T., Letunic, I., Okuda, S., Kanehisa, M. & Bork, P. iPath2. 0: interactive pathway explorer. *Nucleic Acids Res.* **39**, W412-W415 (2011).

8 Zerbino, D. R. & Birney, E. Velvet: algorithms for de novo short read assembly using de Bruijn graphs. *Genome Res.* **18**, 821-829 (2008).

9 Namiki, T., Hachiya, T., Tanaka, H. & Sakakibara, Y. MetaVelvet: an extension of Velvet assembler to de novo metagenome assembly from short sequence reads. *Nucleic Acids Res.* **40**, e155-e155 (2012).

10 Zhu, W., Lomsadze, A. & Borodovsky, M. Ab initio gene identification in metagenomic sequences. *Nucleic Acids Res.* **38**, e132-e132 (2010).

11 Qin, J. *et al.* A metagenome-wide association study of gut microbiota in type 2 diabetes. *Nature* **490**, 55-60 (2012).

Supplementary Figures

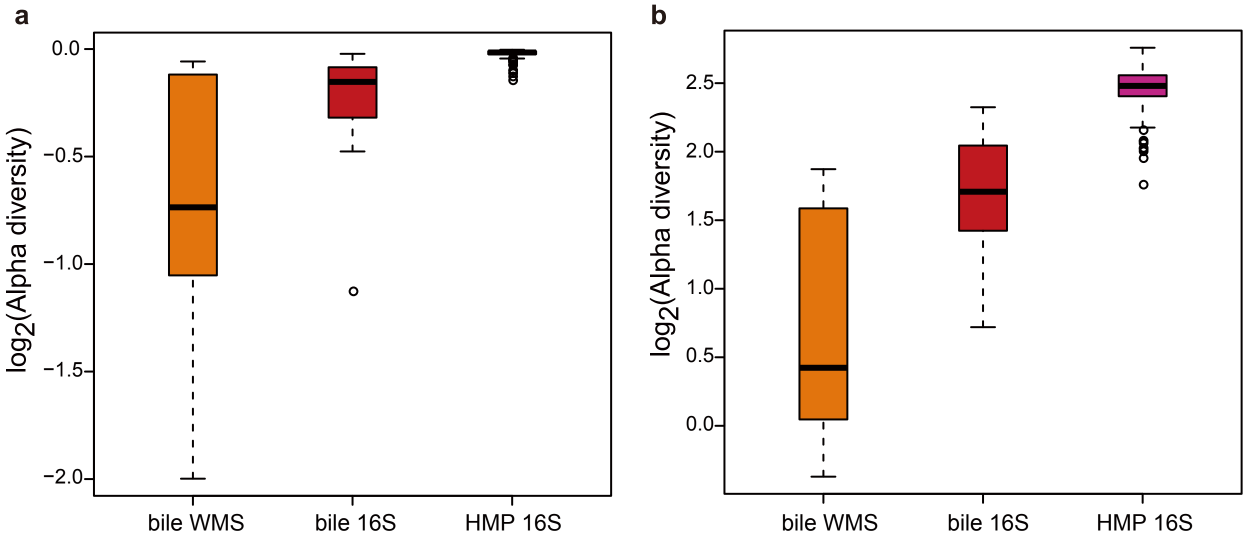

Supplementary Figure S1. Distributions of log2 alpha diversities generated by using Simpson index (a) and Shannon index (b). Orange and red boxplots denote distributions of WMS and 16S sequencing of bile samples respectively, accompanied with the distributions of faecal samples from HMP (purple boxplots). Measured by Simpson index (a) and Shannon index (b), alpha diversities of bile samples based on WMS and 16S sequencing were also significantly lower than those of faecal samples (Wilcoxon rank-sum test, all $P < 5 \times 10^{-9}$). The difference in alpha diversity between bile WMS and 16S sequencing data was also statistically significant (Simpson index, $P = 0.0292$; Shannon index, $P < 0.0023$).

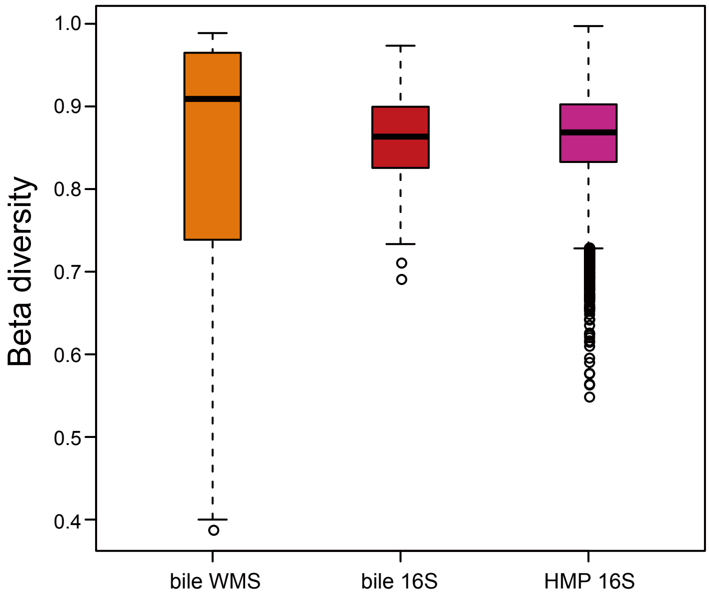

Supplementary Figure S2. Distributions of beta diversities generated by using Jaccard index. Orange and red boxplots denote distributions of WMS and 16S sequencing of bile samples respectively, accompanied with the distributions of faecal samples from HMP (purple boxplots). No significant difference between the beta diversities of bile by 16S sequencing and faecal samples (Wilcoxon rank-sum test, P = 0.18) was observed. Beta diversities of bile by WMS sequencing were slightly higher than those of bile by 16S sequencing (P = 0.044) and of faecal samples (P = 0.02).

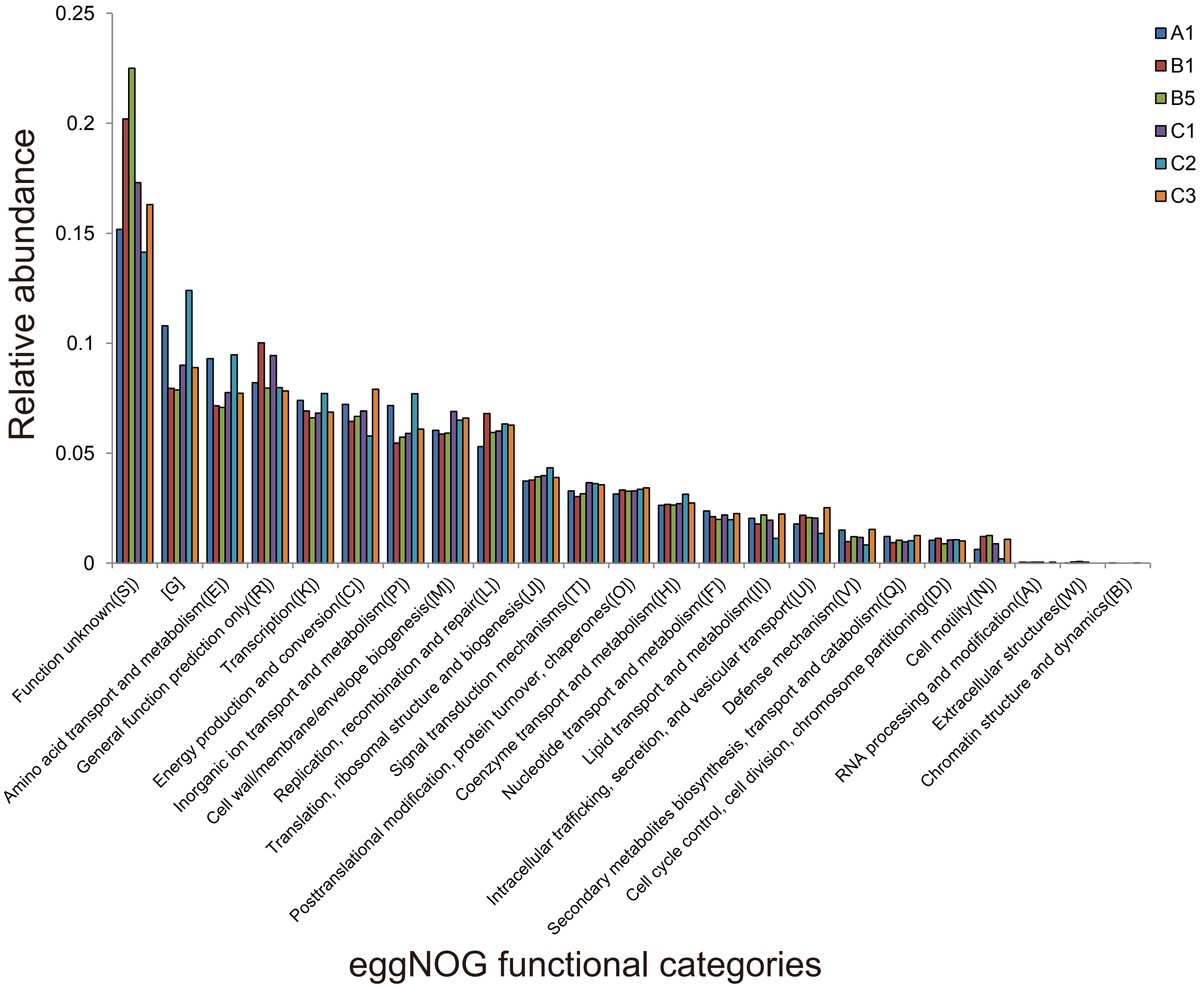

Supplementary Figure S3. Distributions of eggNOG functional categories of the biliary microbiota. Metagenomic assemblies were aligned to eggNOG v4 database. The six samples with >3,500 predicted genes (A1, B1, B5, C1, C2 and C3) were selected for eggNOG functional category analysis. [G]: Carbohydrate transport and metabolism.

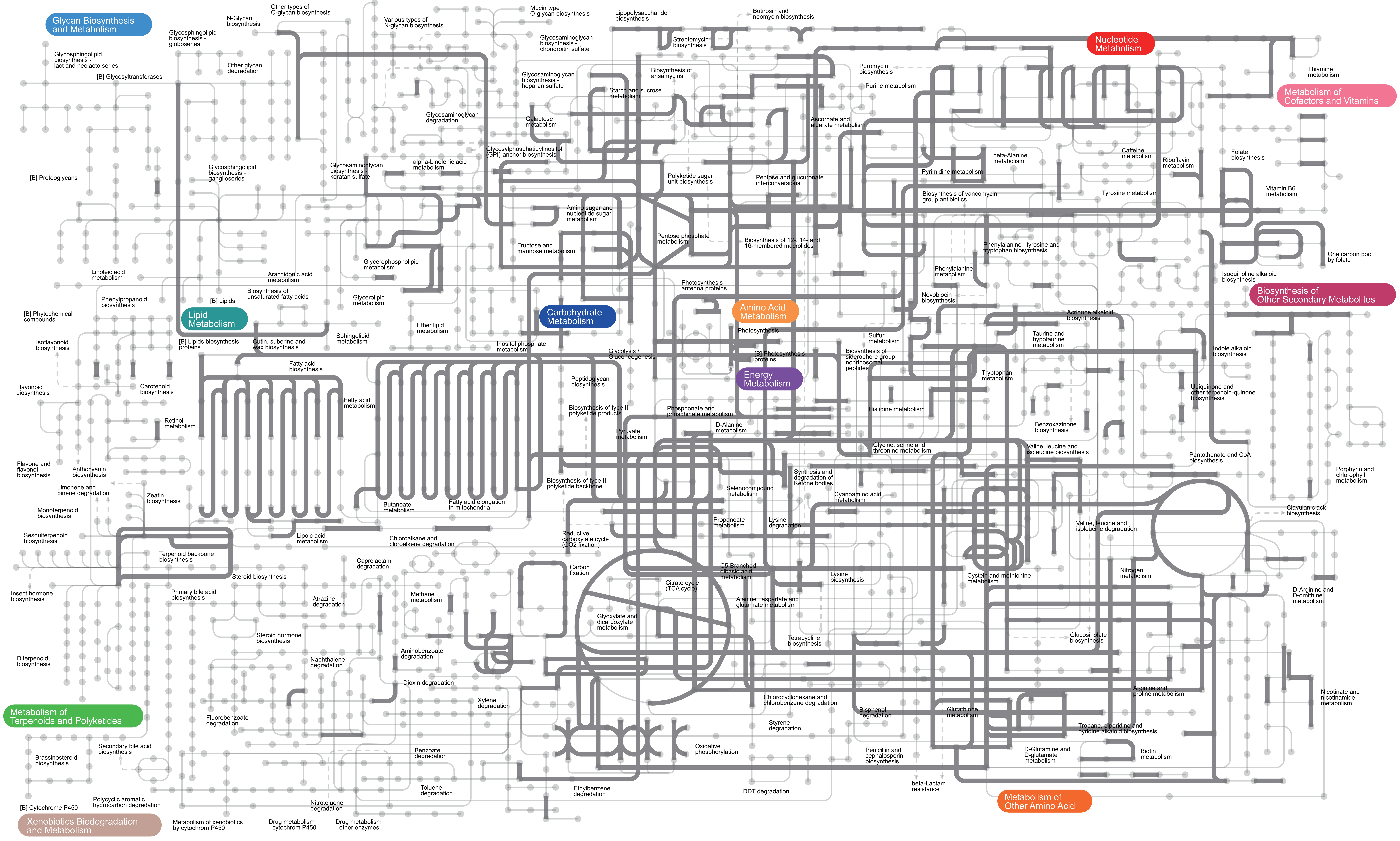

Supplementary Figure S4. Metabolic reconstruction of the biliary microbiota based on eggNOG orthologous groups. EggNOG orthologous groups with ≥0.1% abundance from six samples (A1, B1, B5, C1, C2 and C3) with >3,500 predicted genes were projected to KEGG pathways (bold grey lines) by using iPath v2.0.

Supplementary Tables

Table S2. The origins of bacterial species and the corresponding references.

Species name	Origin	References
Prevotella nigrescens	oral cavity	[1-3]
Neisseria flavescens	oral cavity/upper respiratory tract	[3-5]
Haemophilus parainfluenzae	oral cavity/ respiratory tract/ gastrointestinal tract/ urogenital tract	[3,6,7]
***Veillonella* sp. ACP1**	oral cavity	HMP Reference Genome
Gemella haemolysans	oral cavity/upper respiratory tract	[3,8,9]
Enterococcus faecium	gut	[10]
Fusobacterium periodonticum	oral cavity/gut	[11]
Shigella dysenteriae	gut	[12,13]
***Actinomyces* sp. ICM39**	oral cavity	HMP Reference Genome
Streptococcus salivarius	oral cavity/gut	[14]
Shigella boydii	gut	[12,13]
Neisseria sicca	oral cavity/upper respiratory tract	[3-5]
***Streptococcus* sp. SK140**	oral cavity	HMP Reference Genome
Prevotella nanceiensis	unknown	
Escherichia coli	gut	[6]
Rothia mucilaginosa	oral cavity/ respiratory tract	[15]
Campylobacter rectus	oral cavity	[16]
Prevotella salivae	oral cavity	[3,16,17]
Neisseria meningitidis	oral cavity/upper respiratory tract	[18]
***Prevotella* sp. oral taxon 306**	oral cavity	[3]
***Porphyromonas* sp. oral taxon 279**	oral cavity	[3]
Streptococcus parasanguinis	oral cavity/gut	[4,19]
Megasphaera micronuciformis	oral cavity	[3,11]
Streptococcus pneumoniae	upper respiratory tract	[20,21]
Veillonella atypica	oral cavity/gut	[4,19]
Klebsiella oxytoca	gut	[22]
Klebsiella pneumoniae	oral cavity/skin/gut	[2]
***Streptococcus* sp. I-P16**	unknown	
***Prevotella* sp. C561**	upper respiratory tract	http://www.broadinstitute.org/annotation/genome/Prevotella_group/GenomeDescriptions.html

Streptococcus oralis	oral cavity	[23]
Prevotella veroralis	oral cavity/gut	[19]
Prevotella pallens	oral cavity/gut	[24,25]
Veillonella parvula	oral cavity/gut	[26,27]
Campylobacter concisus	oral cavity/gut	[28]
Prevotella melaninogenica	oral cavity/gut	[1,19]
Streptococcus mitis	oral cavity/pharynx	[29]
Shigella sonnei	gut	[12,13]
Granulicatella adiacens	oral cavity	[25,30]
Fusobacterium nucleatum	oral cavity/gut	[11]
Acinetobacter johnsonii	skin	[31]
Citrobacter freundii	gut	[32]
Enterococcus casseliflavus	oral cavity/gut	[19,33]
***Veillonella* sp. oral taxon 158**	oral cavity	[19,34]
***Shigella* sp. D9**	gut	http://www.broadinstitute.org/annotation/genome/shigella_group/GenomeDescriptions.html
Gemella sanguinis	oral cavity/the upper respiratory/ intestinal tracts	[19,35]
Veillonella dispar	oral cavity/gut	[4]
Streptococcus infantis	oral cavity	[36]
Neisseria subflava	oral cavity/upper respiratory tract	[4]
Shigella flexneri	gut	[12,13]
Neisseria mucosa	oral cavity/upper respiratory tract	[4]
***Enterococcus* sp. C1**	environmental	[37]
Salmonella enterica	gut	[38]
Clostridium perfringens	gut	[39]
Alloprevotella tannerae	oral cavity	[19,40]

References

1 Kuhnert, P., Frey, J., Lang, N. P. & Mayfield, L. Phylogenetic analysis of Prevotella nigrescens, Prevotella intermedia and Porphyromonas gingivalis clinical strains reveals a clear species clustering. *Int. J. Syst. Evol. Microbiol.* **52**, 1391-1395 (2002).

2 Mättö, J. *et al.* Distribution and genetic analysis of oral Prevotella intermedia and Prevotella nigrescens. *Oral Microbiol. Immunol.* **11**, 96-102 (1996).

3 Stewart, L., Grifiss, J. M., Jarvis, G. A. & Way, L. W. Biliary bacterial factors determine the path of gallstone formation. *Am. J. Surg.* **192**, 598-603 (2006).

4 Tronel, H., Chaudemanche, H., Pechier, N., Doutrelant, L. & Hoen, B. Endocarditis due to Neisseria mucosa after tongue piercing. *Clin. Microbiol. Infect.* **7**, 275-276 (2001).

5 Costello, E. K. *et al.* Bacterial community variation in human body habitats across space and time. *Science* **326**, 1694-1697 (2009).

6 Mitchell, J. L. & Hill, S. L. Immune response to Haemophilus parainfluenzae in patients with chronic obstructive lung disease. *Clin. Diagn. Lab. Immunol.* **7**, 25-30 (2000).

7 Lee, D. K., Tarr, P. I., Haigh, W. G. & Lee, S. P. Bacterial DNA in mixed cholesterol gallstones. *Am. J. Gastroenterol.* **94**, 3502-3506 (1999).

8 Lo, W. B., Patel, M., Solanki, G. A. & Walsh, A. R. Cerebrospinal fluid shunt infection due to Gemella haemolysans: Case report. *J. Neurosurg. Pediatr.* **11**, 205-209 (2013).

9 Rose, B., Jeer, P. & Spriggins, A. Gemella haemolysans Infection in Total Hip Arthroplasty. *Case Rep. Orthop.* **2012**, 691703 (2012).

10 Stewart, L., Smith, A. L., Pellegrini, C. A., Motson, R. W. & Way, L. W. Pigment gallstones form as a composite of bacterial microcolonies and pigment solids. *Ann. Surg.* **206**, 242 (1987).

11 Prouty, A., Schwesinger, W. & Gunn, J. Biofilm Formation and Interaction with the Surfaces of Gallstones by Salmonella spp. *Infect. Immun.* **70**, 2640-2649 (2002).

12 Stewart, L., Ponce, R., Oesterk, A. L., Griffiss, J. M. & Way, L. W. Pigment gallstone pathogenesis: slime production by biliary bacteria is more important than beta-glucuronidase production. *J. Gastrointest. Surg.* **4**, 547-553 (2000).

13 Guaglianone, E. *et al.* Microbial biofilms associated with biliary stent clogging. *FEMS Immunol. Med. Microbiol.* **59**, 410-420 (2010).

14 Kaci, G. *et al.* Anti-inflammatory properties of Streptococcus salivarius, a commensal bacterium of the oral cavity and digestive tract. *Appl. Environ. Microbiol.* **80**, 928-934 (2014).

15 Bruminhent, J., Tokarczyk, M. J., Jungkind, D. & DeSimone, J. A. Rothia mucilaginosa prosthetic device infections: a case of prosthetic valve endocarditis. *J. Clin. Microbiol.* **51**, 1629-1632 (2013).

16 Kaufman, H. S., Magnuson, T. H., Lillemoe, K. D., Frasca, P. & Pitt, H. A. The role of bacteria in gallbladder and common duct stone formation. *Ann. Surg.* **209**, 584-592 (1989).

17 Cetta, F. The role of bacteria in pigment gallstone disease. *Ann. Surg.* **213**, 315 (1991).

18 Miller, F. *et al.* Neisseria meningitidis colonization of the brain endothelium and cerebrospinal fluid invasion. *Cell Microbiol.* **15**, 512-519 (2013).

19 Watnick, P. I., Lauriano, C. M., Klose, K. E., Croal, L. & Kolter, R. The absence of a flagellum leads to altered colony morphology, biofilm development and virulence in Vibrio cholerae O139. *Mol. Microbiol.* **39**, 223-235, doi:mmi2195 [pii] (2001).

20 Pericone, C. D., Overweg, K., Hermans, P. W. & Weiser, J. N. Inhibitory and bactericidal effects of hydrogen peroxide production by Streptococcus pneumoniae on other inhabitants of the upper respiratory tract. *Infect. Immun.* **68**, 3990-3997 (2000).

21 Mirza, S. *et al.* Serine protease PrtA from Streptococcus pneumoniae plays a role in the killing of S. pneumoniae by apolactoferrin. *Infect. Immun.* **79**, 2440-2450 (2011).

22 Davey, M. E. & O'Toole G, A. Microbial biofilms: from ecology to molecular genetics. *Microbiol. Mol. Biol. Rev.* **64**, 847-867 (2000).

23 Reichmann, P. *et al.* Genome of Streptococcus oralis strain Uo5. *J. Bacteriol.* **193**, 2888-2889 (2011).

24 Suau, A. *et al.* Direct analysis of genes encoding 16S rRNA from complex communities reveals many novel molecular species within the human gut. *Appl. Environ. Microbiol.* **65**, 4799-4807 (1999).

25 Siqueira, J. F. & Rôças, I. N. Catonella morbi and Granulicatella adiacens: new species in endodontic infections. *Oral Surg. Oral Med. Oral Pathol. Oral Radiol. Endod.* **102**, 259-264 (2006).

26 Rôças, I. & Siqueira, J. Culture-independent detection of Eikenella corrodens and Veillonella parvula in primary endodontic infections. *J. Endod.* **32**, 509-512 (2006).

27 Hughes, C. V., Kolenbrander, P., Andersen, R. & Moore, L. Coaggregation properties of human oral Veillonella spp.: relationship to colonization site and oral ecology. *Appl. Environ. Microbiol.* **54**, 1957-1963 (1988).

28 Kalischuk, L. D. & Inglis, G. D. Comparative genotypic and pathogenic examination of Campylobacter concisus isolates from diarrheic and non-diarrheic humans. *BMC Microbiol.* **11**, 53 (2011).

29 Hohwy, J., Reinholdt, J. & Kilian, M. Population dynamics of Streptococcus mitis in its natural habitat. *Infect. Immun.* **69**, 6055-6063 (2001).

30 Farrell, J. J. *et al.* Variations of oral microbiota are associated with pancreatic diseases including pancreatic cancer. *Gut*, gutjnl-2011-300784 (2011).

31 Seifert, H., Strate, A., Schulze, A. & Pulverer, G. Vascular Catheter—Related Bloodstream Infection Due to Acinetobacter johnsonii (Formerly Acinetobacter calcoaceticus var. lwoffii): Report of 13 Cases. *Clin. Infect. Dis.* **17**, 632-636 (1993).

32 Begley, M., Gahan, C. G. & Hill, C. The interaction between bacteria and bile. *Fems Microbiol. Rev.* **29**, 625-651 (2005).

33 Belzer, C., Kusters, J., Kuipers, E. & Van Vliet, A. Urease induced calcium precipitation by Helicobacter species may initiate gallstone formation. *Gut* **55**, 1678-1679 (2006).

34 Stewart, L., Oesterle, A. L., Erdan, I., Griffiss, J. M. & Way, L. W. Pathogenesis of pigment gallstones in Western societies: the central role of bacteria. *J. Gastrointest. Surg.* **6**, 891-904 (2002).

35 Leung, D. T., Davis, E. M., Qian, Q. & Gold, H. S. First report of prosthetic joint infection by Gemella sanguinis and associated “pseudosatelliting” phenomenon on culture. *J. Clin. Microbiol.* **49**, 3395-3397 (2011).

36 Bek-Thomsen, M., Tettelin, H., Hance, I., Nelson, K. E. & Kilian, M. Population diversity and dynamics of Streptococcus mitis, Streptococcus oralis, and Streptococcus infantis in the upper respiratory tracts of adults, determined by a nonculture strategy. *Infect. Immun.* **76**, 1889-1896 (2008).

37 Swidsinski, A. & Lee, S. P. The role of bacteria in gallstone pathogenesis. *Front. Biosci.* **6**, 93-103 (2001).

38 van den Bogert, B., Boekhorst, J., Smid, E. J., Zoetendal, E. G. & Kleerebezem, M. Draft genome sequence of Veillonella parvula HSIVP1, isolated from the human small intestine. *Genome Announc.* **1**, e00977-00913 (2013).

39 Shimizu, T. *et al.* Complete genome sequence of Clostridium perfringens, an anaerobic flesh-eater. *Proc. Natl. Acad. Sci. U. S. A.* **99**, 996-1001 (2002).

40 O'Toole, G. A. & Kolter, R. Flagellar and twitching motility are necessary for Pseudomonas aeruginosa biofilm development. *Mol. Microbiol.* **30**, 295-304 (1998).

Table S4. Previously reported biliary species identified in our study.

Species	Sample No.[a]	Genus in 16S[b]	Species in 16S[c]	Reference Method[d]	References
Klebsiella pneumoniae	14	Yes	No	cultivation, PCR	[1-16]
Haemophilus parainfluenzae	8	Yes	Yes	cultivation	[17-21]
Escherichia coli	7	Yes	Yes	cultivation,PCR,16S	[1-10,12-16,22-37]
Klebsiella oxytoca	4	Yes	No	cultivation, PCR	[1,6,10-12,14,16]
Enterococcus faecium	4	Yes	No	cultivation	[7,11,14,16,33]
Veillonella parvula	4	Yes	No	cultivation	[38,39]
Clostridium perfringens	3	Yes	Yes	cultivation, PCR	[1,6,14,23-25,32,38,39]
Citrobacter freundii	3	Yes	No	cultivation, PCR	[1,4,6,10,11,14,16,25,33,40]
Enterococcus faecalis	2	Yes	No	cultivation	[11,14-16,33]
Enterobacter cloacae	2	No	No	cultivation, PCR	[1,2,5,6,10,11,14,16,23,33]
Enterobacter aerogenes	2	No	No	cultivation, PCR	[6-8,14,16,23,33,34]
Pseudomonas aeruginosa	2	Yes	No	cultivation	[3,5,9-12,14,16,23,25,26,31,33]
Acinetobacter baumannii	2	Yes	No	cultivation	[11,33]
Rothia mucilaginosa	7	Yes	Yes	cultivation,16S	[37,41]
Propionibacterium acnes	1	Yes	Yes	cultivation,16S	[1,37-39]

[a]The number of the bile samples which contained the species.
[b]The genus that the species belongs to were identified in 16S sequencing at genus level.
[c]The species were identified in 16S sequencing at species level.
[d]The methods were used in references. PCR means species-specific PCR amplification followed by Sanger sequencing. 16S means NGS 16S sequencing.

References

1 Brook, I. Aerobic and anaerobic microbiology of biliary tract disease. *J. Clin. Microbiol.* **27**, 2373-2375 (1989).

2 Karpel, E. *et al.* Bile bacterial flora and its in vitro resistance pattern in patients with acute cholangitis resulting from choledocholithiasis. *Scand. J. Gastroenterol.* **46**, 925-930 (2011).

3 Sahu, M. K., Chacko, A., Dutta, A. K. & Prakash, J. A. J. Microbial profile and antibiotic sensitivity pattern in acute bacterial cholangitis. *Indian J. Gastroenterol.* **30**, 204-208 (2011).

4 Capoor, M. R. *et al.* Microflora of bile aspirates in patients with acute cholecystitis with or without cholelithiasis: a tropical experience. *Braz. J. Infect. Dis.* **12**, 222-225 (2008).

5 Salvador, V. B. D., Lozada, M. C. H. & Consunji, R. J. Microbiology and antibiotic susceptibility of organisms in bile cultures from patients with and without cholangitis at an Asian academic medical center. *Surg. Infect.* **12**, 105-111 (2011).

6 Tabata, M. Bacteria and gallstones. *Dig. Dis. Sci.* **26**, 218-224 (1981).

7 Ohdan, H. *et al.* Bacteriological investigation of bile in patients with cholelithiasis. *Surg. Today* **23**, 390-395 (1993).

8 Hochwald, S. N., Burke, E. C., Jarnagin, W. R., Fong, Y. & Blumgart, L. H. Association of preoperative biliary stenting with increased postoperative infectious complications in proximal cholangiocarcinoma. *Arch. Surg.* **134**, 261-266 (1999).

9 Jagannath, P. *et al.* Effect of preoperative biliary stenting on immediate outcome after pancreaticoduodenectomy. *Br. J. Surg.* **92**, 356-361 (2005).

10 Rerknimitr, R. *et al.* Microbiology of bile in patients with cholangitis or cholestasis with and without plastic biliary endoprosthesis. *Gastrointest. Endosc.* **56**, 885-889 (2002).

11 Yu, H. *et al.* Bile culture and susceptibility testing of malignant biliary obstruction via PTBD. *Cardiovasc. Interv. Radiol.* **35**, 1136-1144 (2012).

12 Brody, L. A. *et al.* Clinical factors associated with positive bile cultures during primary percutaneous biliary drainage. *J. Vasc. Interv. Radiol.* **9**, 572-578 (1998).

13 Davey, M. E. & O'Toole G, A. Microbial biofilms: from ecology to molecular genetics. *Microbiol. Mol. Biol. Rev.* **64**, 847-867 (2000).

14 Pratt, L. A. & Kolter, R. Genetic analysis of Escherichia coli biofilm formation: roles of flagella, motility, chemotaxis and type I pili. *Mol. Microbiol.* **30**, 285-293 (1998).

15 Swidsinski, A. & Lee, S. P. The role of bacteria in gallstone pathogenesis. *Front. Biosci.* **6**, 93-103 (2001).

16 Lo, W. B., Patel, M., Solanki, G. A. & Walsh, A. R. Cerebrospinal fluid shunt infection due to Gemella haemolysans: Case report. *J. Neurosurg. Pediatr.* **11**, 205-209 (2013).

17 Neu, A. M., Case, B., Lederman, H. M. & Fivush, B. A. Neisseria sicca peritonitis in a patient maintained on chronic peritoneal dialysis. *Pediatr. Nephrol.* **8**, 601-602 (1994).

18 Bruminhent, J., Tokarczyk, M. J., Jungkind, D. & DeSimone, J. A. Rothia mucilaginosa prosthetic device infections: a case of prosthetic valve endocarditis. *J. Clin. Microbiol.* **51**, 1629-1632 (2013).

19 Miller, F. *et al.* Neisseria meningitidis colonization of the brain endothelium and cerebrospinal fluid invasion. *Cell Microbiol.* **15**, 512-519 (2013).

20 Pericone, C. D., Overweg, K., Hermans, P. W. & Weiser, J. N. Inhibitory and bactericidal effects of hydrogen peroxide production by Streptococcus pneumoniae on other inhabitants of the upper respiratory tract. *Infect. Immun.* **68**, 3990-3997 (2000).

21 Mirza, S. *et al.* Serine protease PrtA from Streptococcus pneumoniae plays a role in the killing of S. pneumoniae by apolactoferrin. *Infect. Immun.* **79**, 2440-2450 (2011).

22 Flemma, R. J., Flint, L. M., Osterhout, S. & Shingleton, W. W. Bacteriologic studies of biliary tract infection. *Ann. Surg.* **166**, 563 (1967).

23 England, D. M. & Rosenblatt, J. Anaerobes in human biliary tracts. *J. Clin. Microbiol.* **6**, 494-498 (1977).

24 Shimada, K., Inamatsu, T. & Yamashiro, M. Anaerobic bacteria in biliary disease in elderly patients. *J. Infect. Dis.* **135**, 850-854 (1977).

25 Leung, J. W. *et al.* Bacteriologic analyses of bile and brown pigment stones in patients with acute cholangitis. *Gastrointest. Endosc.* **54**, 340-345 (2001).

26 Abeysuriya, V., Deen, K. I., Wijesuriya, T. & Salgado, S. S. Microbiology of gallbladder bile in uncomplicated symptomatic cholelithiasis. *Hepatobiliary Pancreat. Dis. Int.* **7**, 633-637 (2008).

27 Stewart, L., Oesterle, A. L., Erdan, I., Griffiss, J. M. & Way, L. W. Pathogenesis of pigment gallstones in Western societies: the central role of bacteria. *J. Gastrointest. Surg.* **6**, 891-904 (2002).

28 Kanafani, Z. A. *et al.* Antibiotic use in acute cholecystitis: practice patterns in the absence of evidence-based guidelines. *J. Infect.* **51**, 128-134 (2005).

29 Van Leeuwen, P., Keeman, J., Butzelaar, R. & Van den Bogaard, A. Correlation between a positive gallbladder culture and subsequent wound infection after biliary surgery--a retrospective study of 840 patients. *Neth. J. Surg.* **37**, 179-182 (1985).

30 Den Hoed, P., Boelhouwer, R., Veen, H., Hop, W. & Bruining, H. Infections and bacteriological data after laparoscopic and open gallbladder surgery. *J. Hosp. Infect.* **39**, 27-37 (1998).

31 Al‐Abassi, A. A., Farghaly, M. M., Ahmed, H. L., Mobasher, L. L. & Al‐Manee, M. S. Infection after laparoscopic cholecystectomy: effect of infected bile and infected gallbladder wall. *Eur. J. Surg.* **167**, 268-273 (2001).

32 Povoski, S. P., Karpeh Jr, M. S., Conlon, K. C., Blumgart, L. H. & Brennan, M. F. Preoperative biliary drainage: impact on intraoperative bile cultures and infectious morbidity and mortality after pancreaticoduodenectomy. *J. Gastrointest. Surg.* **3**, 496-505 (1999).

33 Cortes, A. *et al.* Effect of bile contamination on immediate outcomes after pancreaticoduodenectomy for tumor. *J. Am. Coll. Surg.* **202**, 93-99 (2006).

34 Volpi, L. *et al.* A rare localization of actinomycosis mimicking ulcerative malignancy. *Case. Rep. Otolaryngol.* **2013**, 323210 (2013).

35 Swidsinski, A., Khilkin, M., Pahlig, H., Swidsinski, S. & Priem, F. Time dependent changes in the concentration and type of bacterial sequences found in cholesterol gallstones. *Hepatology* **27**, 662-665 (1998).

36 Lee, D. K., Tarr, P. I., Haigh, W. G. & Lee, S. P. Bacterial DNA in mixed cholesterol gallstones. *Am. J. Gastroenterol.* **94**, 3502-3506 (1999).

37 Wu, T. *et al.* Gut microbiota dysbiosis and bacterial community assembly associated with cholesterol gallstones in large-scale study. *BMC Genomics* **14**, 669 (2013).

38 Qin, N. *et al.* Alterations of the human gut microbiome in liver cirrhosis. *Nature* **513**, 59-64 (2014).

39 Kaya, D., Demirezen, Ş., Hasçelik, G., Kıvanç, D. G. & Beksaç, M. S. Comparison of PCR, culturing and Pap smear microscopy for accurate diagnosis of genital Actinomyces. *J. Med. Microbiol.* **62**, 727-733 (2013).

40 Sudo, T. *et al.* Specific antibiotic prophylaxis based on bile cultures is required to prevent postoperative infectious complications in pancreatoduodenectomy patients who have undergone preoperative biliary drainage. *World J.Surg.* **31**, 2230-2235 (2007).

41 Mitchell, J. L. & Hill, S. L. Immune response to Haemophilus parainfluenzae in patients with chronic obstructive lung disease. *Clin. Diagn. Lab. Immunol.* **7**, 25-30 (2000).
